# Envelope Proteins of White Spot Syndrome Virus (WSSV) Interact with *Litopenaeus vannamei* Peritrophin-Like Protein (LvPT)

**DOI:** 10.1371/journal.pone.0144922

**Published:** 2015-12-21

**Authors:** Shijun Xie, Xiaojun Zhang, Jiquan Zhang, Fuhua Li, Jianhai Xiang

**Affiliations:** 1 Key Laboratory of Experimental Marine Biology, Institute of Oceanology, Chinese Academy of Sciences, Qingdao, 266071, China; 2 University of Chinese Academy of Sciences, Beijing, 100049, China; St. Jude Children's Research Hospital, UNITED STATES

## Abstract

White spot syndrome virus (WSSV) is a major pathogen in shrimp cultures. The interactions between viral proteins and their receptors on the surface of cells in a frontier target tissue are crucial for triggering an infection. In this study, a yeast two-hybrid (Y2H) library was constructed using cDNA obtained from the stomach and gut of *Litopenaeus vannamei*, to ascertain the role of envelope proteins in WSSV infection. For this purpose, VP37 was used as the bait in the Y2H library screening. Forty positive clones were detected after screening. The positive clones were analyzed and discriminated, and two clones belonging to the peritrophin family were subsequently confirmed as genuine positive clones. Sequence analysis revealed that both clones could be considered as the same gene, LV-peritrophin (*LvPT*). Co-immunoprecipitation confirmed the interaction between LvPT and VP37. Further studies in the Y2H system revealed that LvPT could also interact with other WSSV envelope proteins such as VP32, VP38A, VP39B, and VP41A. The distribution of *LvPT* in tissues revealed that *LvPT* was mainly expressed in the stomach than in other tissues. In addition, LvPT was found to be a secretory protein, and its chitin-binding ability was also confirmed.

## Introduction

The white spot syndrome virus (WSSV) is a major pathogen that infects shrimp in cultures. It is an enveloped virus with a 300-kb circular double-strand DNA [1,2] that infects a wide range of decapod and non-decapod crustacean hosts in natural environments [3]. Several envelope proteins produced by WSSV, such as VP19, VP28, VP37, and VP466, have been previously identified [4–6]. Initial encounters between the virus and its host cell are mediated through viral surface components such as membrane glycoproteins, or sites on a viral capsid [7]. These receptors function as keys, allowing the fusion of a virus with a cell, or its attachment to a cell. Previous studies [8,9] have reported that WSSV infects shrimps through oral ingestion. The digestive epithelial cells in the midgut of the *Marsupenaeus japonicus* trunk was considered to be the transient infection site, which allowed the WSSV to cross the underlying basal lamina [9]. Therefore, the cells of the stomach and midgut appear to be important factors influencing the WSSV infection; however, the exact factors involved in WSSV infection remain to be determined.

The yeast two-hybrid (Y2H) system is a powerful method for the detection and analysis of protein-protein interactions [10]. Many interactions between viruses such as BmNPV [11], BmDNV [12], and CSFV [13] and their hosts have been detected using the Y2H system.

In this work, a Y2H library was constructed using cDNA synthesized from the genetic material of the stomach and gut of *L*. *vannamei* to elucidate the mechanism of WSSV infection in the host stomach and midgut. For this purpose, the VP37 envelope protein was used as bait in the Y2H screening. Subsequently, the interactions between the prey protein of interest and other WSSV envelope proteins in Y2H system were also analyzed.

## Materials and Methods

### Experimental animals and collection of tissues

Adult shrimp (*L*. *vannamei*) sized approximately 18 cm were used for the construction of the Y2H library; these were obtained from the Hainan Province of China. Legs were extracted from 10 shrimp for DNA extraction prior to the construction of the library. All DNA samples were analyzed to detect WSSV infection by polymerase chain reaction (PCR), using the primers VP28-F (5′-ATGGATCTTTCTTTCACTCTTTC-3′) and VP28-R (5′-CTCGGTCTCAGTGCCAG-3′). The stomach and guts dissected from two individuals that did not display a positive VP28 signal were used to construct the Y2H library.

The distribution of *LvPT* in different tissues was studied in five individuals (approximately 11 cm) that were cultured in the laboratory from the post-larval stage. The heart, hepatopancreas, gill, lymphoid organ, muscle, gut, stomach, and skin were dissected from each individual and subsequently pooled. Each intact gut was equally divided into three parts (length-wise) during dissection; these were named gut1, gut2, and gut3 along the anterior-posterior axis.

Both of the adult shrimp used for library construction and tissue distribution in this study came from a variety of shrimp named Kehai No.1 [14]. This variety was selected and bred by our laboratory cooperated with local company in Hainan province.

### Construction of cDNA and Y2H library

The cDNA and Y2H libraries were constructed in collaboration with Shanghai OE Biotech. Total RNA was extracted from the stomach and gut using TRIzol (Invitrogen, Carlsbad, CA, USA); mRNA was isolated using the FastTrack^®^ MAG mRNA isolation Kit (Invitrogen). cDNA was synthesized using the CloneMiner II cDNA Library Construction Kit (Invitrogen), according to the protocols provided by the manufacturer. Three forward adapters (listed in Table 1) were separately ligated to the 5′-end of the cDNA after the synthesis of second cDNA strand in order to ensure that the cDNA was translated in the right reading frame.

**Table 1:** Adapters used in the synthesis of cDNA.

adapter	sequence (5′→3′)
attB1 RFα	TCGTCGGGGACAACTTTGTACAAAAAAGTTGG
attB1 RFβ	TCGTCGGGGACAACTTTGTACAAAAAAGTTGGA
attB1 RFγ	TCGTCGGGGACAACTTTGTACAAAAAAGTTGGAA
attB2	ACCCAGCTTTCTTGTACAAAGTGGT

**Fig 1:**
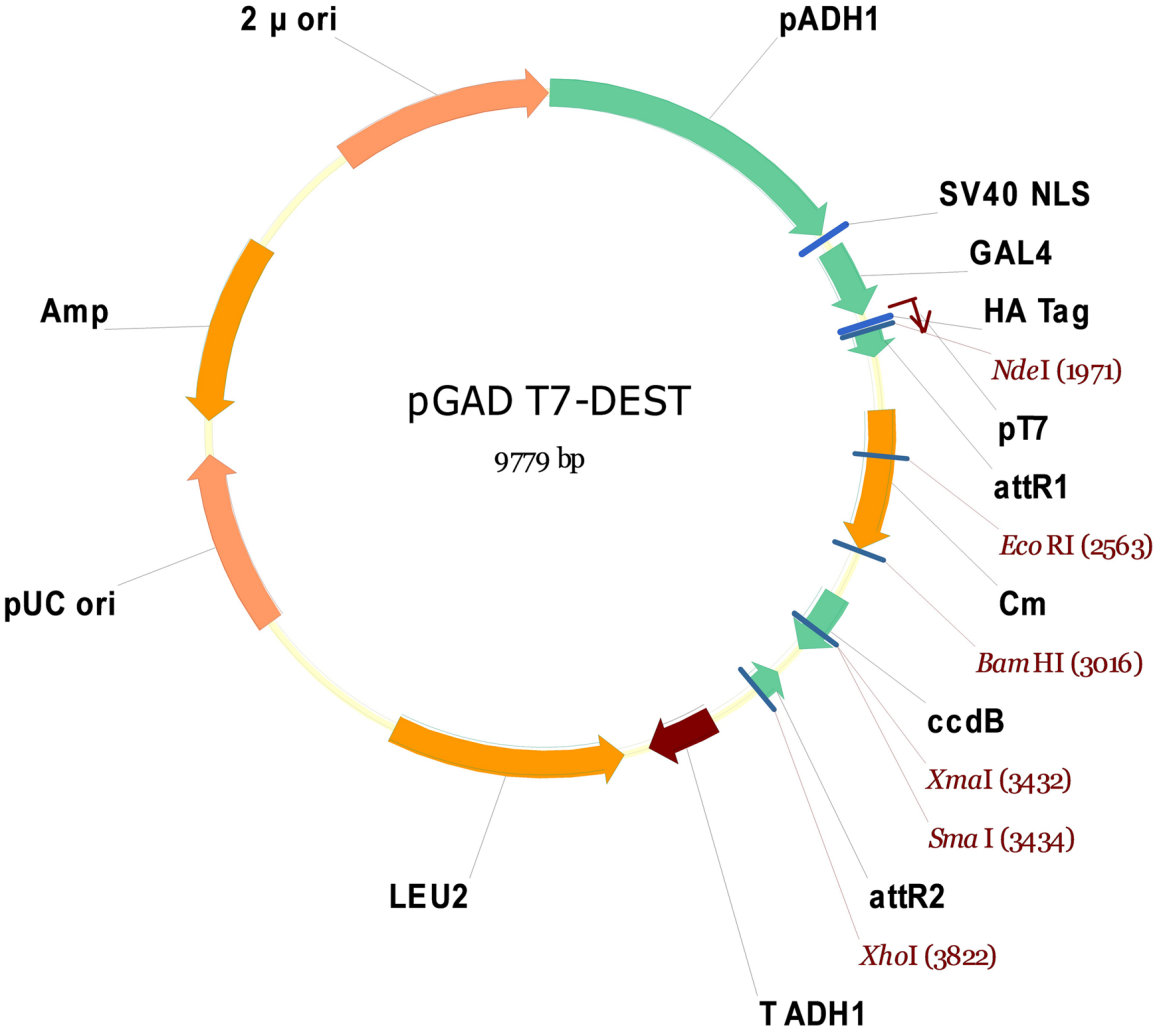
Map of pGADT7-DEST. This plasmid was modified from pGADT7-Rec (Clontech Laboratories); the SMART III and CDS III sequences were replaced by attR1 and attR2, respectively. Following recombination catalysis using the Gateway^®^ LR Clonase^™^ II enzyme mix (Invitrogen), attR1-Cm-ccdB-attR2 was replaced by attB1-cDNA insert-attB2.

Purified cDNA ligated with three different forward adapters were mixed and recombined with the pDONR^™^222 plasmid vector via catalysis, using the Gateway^®^ BP Clonase^™^ II Enzyme Mix (Invitrogen). The recombination product was transformed into *E*. *coli* (DH10B) by electroporation, in order to form the cDNA library. The quality of the cDNA library was evaluated, and the *E*. *coli* (DH10B) containing the cDNA library was cultured in broth medium; subsequently, the cDNA library plasmid was extracted. The cDNA library plasmid was recombined with modified pGADT7-Rec (or pGADT7- DEST; Fig 1) using the Gateway^®^ LR Clonase^™^ II enzyme mix (Invitrogen). The product of the second recombination was transformed into *E*. *coli* (DH10B) by electroporation, in order to create the Y2H library. The quality of the Y2H library was evaluated, *E*. *coli* (DH10B) containing the Y2H library was cultured in broth medium, and the Y2H library plasmid was subsequently extracted. The Y2H library plasmid was transformed into yeast Y187 (Clontech Laboratories, USA) according to the user manual of the Yeastmaker^™^ Yeast Transformation System 2 (Clontech Laboratories). The quality of the Y2H library in Y187 was evaluated, and the library was subsequently stored at -80°C until use.

### Construction of the bait strain

Previous studies have indicated that the full-length VP37 protein could autonomously activate reporter genes in the absence of the prey protein; therefore, a truncated C-terminal VP37 (aa 1–247) was constructed in the present work. The truncated C-terminal VP37 was amplified from the WSSV genome by using forward and reverse primers containing the *Nde*I and *Pst*I restriction sites: VP37Y-F (5′- TTCGGCATATGGCGGTAAACTTGGAT-3′) and VP37Y-R (5′-TCACTGCAGTCTGTTGTTTTCAGCGACAC-3′). The VP37 bait vector (pGBKT7-37) was constructed using the appropriate restriction enzyme (TaKaRa Bio Inc., Otsu, Japan), in order to clone the VP37 genes into pGBKT7 (Clontech Laboratories). The pGBKT7-37 was sequenced prior to transformation to ensure that the bait protein (VP37) encodes in the correct reading frame of the GAL4 DNA binding domain; pGBKT7-37 was subsequently transformed into the Y2HGold yeast strain (Clontech Laboratories) according to the user manual of the Yeastmaker^™^ Yeast Transformation System 2 (Clontech Laboratories). The toxicity and auto-activation of bait protein were examined according to user manual of the Matchmaker^™^ Gold Yeast Two-Hybrid System (Clontech Laboratories).

### Y2H library screening

The obtained Y2H library was screened using the Matchmaker^™^ Gold Yeast Two-Hybrid System (Clontech Laboratories) as per the manufacturer’s instructions. The VP37 bait strain was cultured overnight in a shaker (30°C, 250 rpm) in 50 mL SD/-Trp liquid medium until the optical density at 600 nm (OD_600_) met the requirement (0.8) for Y2H screening. The VP37 bait strain was collected and combined with 1 mL of the library strain in a 2-L flask, and cultured in 45 mL 2x YPDA liquid medium supplemented with 50 μg/mL kanamycin at 30°C and 50 rpm shaking for 24 h. The mated culture was subsequently analyzed under a microscope to confirm the presence or absence of zygotes. The mated culture was then spread on double dropout medium (SD/-Leu/-Trp supplemented with X-a-Gal and Aureobasidin A; DDO/X/A) plates. After a few days, all clones that showed growth on the DDO/X/A plates were patched on to plates containing quadruple dropout medium (SD/-Ade/-His/-Leu/-Trp supplemented with X-a-Gal and Aureobasidin A; QDO/X/A). Finally, all positive clones grown on QDO/X/A plates were picked and cultured in quadruple dropout liquid medium (QDO; SD/-Ade/-His/-Leu/-Trp) overnight in a shaker (30°C, 250 rpm). Subsequently, the bait and prey plasmids were isolated using the Easy Yeast Plasmid Isolation Kit (Clontech Laboratories). These plasmids were subjected to further analyses, including sequencing and prey plasmid rescue.

### Confirmation of positive clones of interest

The interactions between prey and bait proteins were confirmed using the Matchmaker^™^ Gold Yeast Two-Hybrid System (Clontech Laboratories) as per the protocols detailed by the manufacturer. The prey plasmids pGBKT7-37 and pGBKT7 were transformed into the yeast Y2HGold strain (Clontech Laboratories), which was subsequently cultured in DDO plates. Clones grown on DDO plates were subsequently patched onto QDO/X/A plates. Positive clones (showing growth on QDO/X/A plates) were sequenced in order to confirm that the prey could encode in the reading frame containing the GAL4 DNA activation domain. Positive (pGBKT7-53/pGADT7-T) and negative (pGBKT7-Lam/pGADT7-T) controls were also prepared.

### Sequence analysis

Clones that gave a genuine positive confirmation were further analyzed. Sequences were translated into deduced amino acid sequences in the right reading frame. The deduced amino acid sequences of the positive clones were analyzed using Protein Basic Local Alignment Search Tool (BLASTP) (http://blast.ncbi.nlm.nih.gov/blast). The molecular weight (MW) and pI of the deduced amino acids were predicted using the ExPaSy server (http://web.expasy.org/compute_pi/). Protein domains were detected using the SMART program (http://smart.embl-heidelberg.de/), and multiple sequence alignments were performed using Clustal Omega (http://www.ebi.ac.uk/Tools/msa/clustalo/).

### Distribution of *LvPT* in different tissues

Total RNA was extracted from 10 tissues by using RNAiso Plus (TaKaRa Bio Inc.) according the manufacturer’s instructions. The extracted RNA was quantified using the Nanodrop ND2000 spectrophotometer (Thermo Fisher Scientific, Waltham, MA, USA). Total RNA was reverse transcribed into cDNA using the *TransScript* ^®^ One-Step gDNA Removal and cDNA Synthesis SuperMix (TransGen Biotech, China). The relative level of expression of *LvPT* was analyzed by semi-quantitative PCR, using 18S rRNA as the reference gene. The nucleoside sequence identity between the two clones was 98%; therefore, these two clones were considered to belong to the same gene (LV-peritrophin; *LvPT*). Subsequently, only one pair of primers was used: PTRT-F (5′-TGTGCAAGCAGGAGGGCCGATAT-3′), PTRT-R (5′-CTGGACAGAAGCCGTCAGGAATG-3′), 18S rRNA-F (5′-TATACGCTAGTGGAGCTGGAA-3′), and 18S rRNA-R (5′-GGGGAGGTAGTGACGAAAAAT-3′). The expected *LvPT* and 18S rRNA fragment lengths were 222 and 166 bps, respectively. The PCR for 18S rRNA was performed using the following reaction conditions: 20 cycles of 95°C for 15 s, 57°C for 15 s, and 72°C for 20 s. The quantification of the *LvPT* gene was performed using the following PCR conditions: 26 cycles of 95°C for 15 s, 59°C for 15 s, and 72°C for 20 s.

### Expression plasmid construction and transfection into Sf9 cells

The full length of the *LvPT* gene was amplified using the primers pDHspPTF and pDHspPTR; *LvPT* without the signal peptide, or *LvPT* ^*WSP*^, was amplified using the primers pDHspPT^WSP^F and pDHspPTR, while *VP37* was amplified using the primers pDHspVP37F and pDHspVP37R. *LvPT* and *LvPT* ^*WSP*^ were inserted in the pDHsp/V5-His vector through the appropriate restriction enzyme sites (*BamHI* and *EcoRI*), while *VP37* was inserted into pDHsp/FLAG-His (both plasmids were generously provided by Pro Lo [15], generating plasmids pDHsp/LvPT-V5-His, pDHsp/LvPT^WSP^-V5-His, and pDHsp/VP37-FLAG-His. All primers used in the construction of expression plasmids were listed in Table 2.

**Table 2:** Primers used in the construction of expression plasmids.

Primer	Sequence (from 5′ to 3′)
pDHspPTF	GGATCCATGAGGTCCAATACGTTCT
pDHspPTR	GAATTCTCTGGACAGAAGCCGTCAGGAAT
pDHspPT^WSP^F	GGATCCATGAGAGACTTGCGAGCTAAAC
pDHspVP37F	GGATCCATGGCGGTAAACTTGGAT
pDHspVP37R	GAATTCGTCCAACAATTTAAAAAGAAGTAGA

Sf9 cells were seeded onto a 6-well plate (1.2 × 10^6^ cells per well) containing Sf-900^™^ III SFM (Gibco, USA) 15 min before transfection. The plasmids were transfected using Lipofectamine^®^ 2000 (Invitrogen, USA); Opti-MEM^®^ I (Gibco, USA) was used to dilute the plasmid and transfection reagent. The plasmids (8 μg) were transfected into the Sf9 cells in each well. Medium containing the DNA-lipid complex was replaced by fresh Sf-900^™^ III SFM medium 6 h after transfection. These were cultured overnight at 27°C; the cells were then subjected to heat-shock treatment (42°C for 30 min), and subsequently cooled to 27°C. The cells are ready for protein extraction 6 h after the heat shock treatment.

### Co-immunoprecipitation

Sf9 cells were transfected with different plasmids (pDHsp/LvPT-V5-His and pDHsp/VP37-FLAG-His, pDHsp/-V5-His and pDHsp/VP37-FLAG-His, pDHsp/LvPT-V5-His and pDHsp/FLAG-His) and subjected to heat shock treatment. The cells in each well were lysed in PBS lysis buffer (0.1 M, pH 6.0, 1% NP-40; 120 μL) supplemented with a protease inhibitor cocktail tablet (Sangon Biotech, China). The cells were lysed at 4°C for 10 min with occasional shaking. The lysate was centrifuged at 12,000 rpm for 2 min and the supernatant (10 μL) was used to confirm the expression of LvPT-V5 and VP37-FLAG. All samples were separated by 10% sodium dodecyl sulfate polyacrylamide gel electrophoresis (SDS-PAGE) and subsequently transferred to polyvinylidene difluoride (PVDF) membranes. The PVDF membranes were incubated with blocking buffer (5% no fat milk in TBS [50 Mm Tris-HCl, pH 8.0; 150 Mm NaCl]) at room temperature (25°C) for 2 h, and subsequently incubated with blocking buffer containing horseradish peroxidase (HRP)-conjugated anti-His antibody (CW Biotech, China) for 1 h at room temperature. The membrane was then washed thrice with 1% Tween 20 in TBS (TBST); the HRP-conjugated anti-His antibody was detected using a commercial HRP-DAB chromogenic substrate kit (Tiangen, China). Co-immunoprecipitation was performed according to a previously described procedure [15]. The supernatant (80 μL) was then incubated overnight in an 8 μL anti-FLAG M2 affinity gel (Sigma-Aldrich, USA) at 4°C overnight with rotation. The gel was then washed twice in 100 μL PBS lysis buffer. The presence of LvPT-V5 and VP37-FLAG in the immunoprecipitated complexes was detected as described above.

### Expression of LvPT and LvPT^WSP^ in Sf9 cells and chitin binding assay

Sf9 cells were transfected with the appropriate plasmids (pDHsp/LvPT-V5-His and pDHsp/LvPT^WSP^-V5-His), and were subsequently subjected to heat shock treatment. The culture medium supernatant was collected 6 h after the heat shock treatment; the cells in each well were lysed with 150 μL TBS lysis buffer (50 Mm Tris-HCl, pH 8.0; 150 Mm NaCl; and 1% NP-40) supplemented with a protease inhibitor cocktail tablet at 4°C for 10 min with occasional shaking. The lysate was centrifuged at 12,000 rpm for 2 min, and the supernatant and precipitate were collected. The supernatant of the culture medium, lysate, and precipitate were used to confirm the expression of LvPT-V5 and LvPT^WSP^-V5. The supernatant of the culture medium containing LvPT-V5 was used to perform a chitin binding assay. Four hundred microliters 4% (W/V) colloidal chitin (in each of nine tubes) was centrifuged at 12,000 rpm for 5 min; the supernatant was discarded, and the precipitate washed with 400 μL TBS. This step was repeated thrice for each tube. The culture medium supernatants (150 μL) were added to one tube each (four tubes in total), while 150 μL bovine serum albumin (BSA; 1 mg/mL) was added to four tubes as the negative control. The remaining tube (without any protein) was used as the blank. All tubes were incubated at 4°C for 2 h with rotation, and the protein-chitin complex was subsequently washed thrice with TBS (50 Mm Tris, pH 8.0) supplemented with 50 mM NaCl, 100 mM NaCl, 200 mM NaCl, and 500 mM NaCl. The protein-chitin complex in each tube was then dissociated using 30 μL of the SDS-PAGE loading buffer (TaKaRa). LvPT-V5 protein expression was detected by western blot, using a HRP-conjugated anti-His antibody. The BSA was detected by SDS-PAGE.

### Interaction between LvPT and WSSV envelope proteins

The interactions between LvPT and other envelope proteins of WSSV were explored by amplifying the *VP19*, *VP24*, *VP28*, *VP32*, *VP38A*, *VP39B*, *VP41A*, *VP41B*, *VP51B*, *VP60A*, and *VP73* genes from the WSSV genome, utilizing the primers listed in Table 3. This amplified genes were cloned into the pGBKT7 vector (Clontech Laboratories) using a restriction enzyme (TaKaRa). The toxicity and auto-activation of each envelope protein were separately examined. Each envelope protein and prey plasmid pair was co-transformed into the yeast Y2HGold strain (Clontech Laboratories), which was subsequently cultured in DDO plates. Clones that showed growth on the DDO plates were then patched onto QDO/X/A plates. Positive (pGBKT7-53/pGADT7-T) and negative (pGBKT7-Lam/pGADT7-T) controls were also prepared.

**Table 3:** Primers used in the construction of the Y2H plasmid.

primer	sequence (from 5′ to 3′)
VP19Y-F	CATATGATGGCCACCACGACTAACACT
VP19Y-R	GGATCCTTACTGCCTCCTCTTGGGGTG
VP24Y-F	CATATGATGGAATTTGGCAACCTAAC
VP24Y-R	GGATCCTTACTTCTTCTTGATTTCGTCCT
VP28Y-F	GGCCATATGATGGATCTTTCTTTCACTCTTTC
VP28Y-R	GGATCCTTACTCGGTCTCAGTGCCAG
VP32Y-F	CATATGATGTCTTTGTTTATTTCCCCCTT
VP32Y-R	GGATCCTCAACGGAGACTAGATCCACGG
VP38AY-F	CATATGATGTCTTCTTCGTCTTCTGAAACTC
VP38AY-R	GGATCCTTATGAACATGTTACAATTATTCGA
VP39BY-F	CATATGATGTCGTCTAACGGAGATGAGCCTG
VP39BY-R	GGATCCCTAAAAAACAAACAGATTGAAAT
VP41AY-F	CATATGATGTTGTTTGATTTCTTTCTAA
VP41AY-R	CTGCAGCTATATAATACGGGACCTGACG
VP41BY-F	GAATTCATGGGAGATAAGCAAAAGGTGG
VP41BY-R	CTGCAGCTAGGAGCATGTGCATGTGATC
VP51BY-F	CCATGGAGATGATATTTTATACAATGCAACCCT
VP51BY-R	GGATCCTTAATCGTCTACCAAATTTTCTGTT
VP60AY-F	CATATGATGGCTGTAGGGGATTATCTTTCT
VP60AY-R	GGATCCTTACACAATTTTCTTTATTTTTTCT
VP73Y-F	CCATGGAGATGGCAGGGAATAGAACCCAGTT
VP73Y-R	GGATCCTTATACGGGAAAATTCTCCAGAAGG

## Results

### Detection of WSSV infection in shrimp

**Fig 2:**
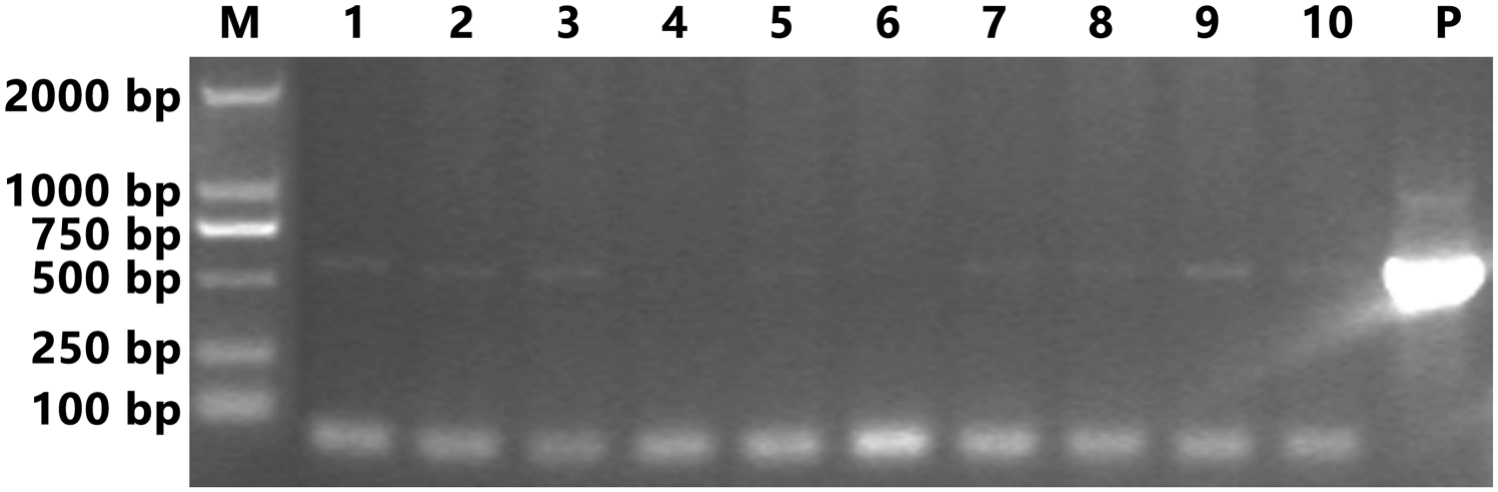
Agarose gel bands of PCR-amplified DNA fragments obtained from the leg of each shrimp (n = 10). Lane M contained the D2000 II DNA ladder (Real-Times Biotechnology, Beijing, China). Lanes 1–10 contained the amplified DNA fragments from 10 different shrimps (marked No. 1 to 10). Lane P displays the *VP28* positive control. The expect size of positive band was around 621 bps.

40 cycles (94°C for 30 s, 50°C for 30 s, and 72°C for 1 min) in order to ensure the amplification of *VP28* from potential WSSV-infected shrimp. The amplified fragments were analyzed by agarose gel (1%) electrophoresis, and the resultant gel is shown in Fig 2. Shrimp 4 and 6 were considered to be VP28-negative and were therefore used for the construction of the Y2H library.

### Y2H library quality and screening results

**Fig 3:**
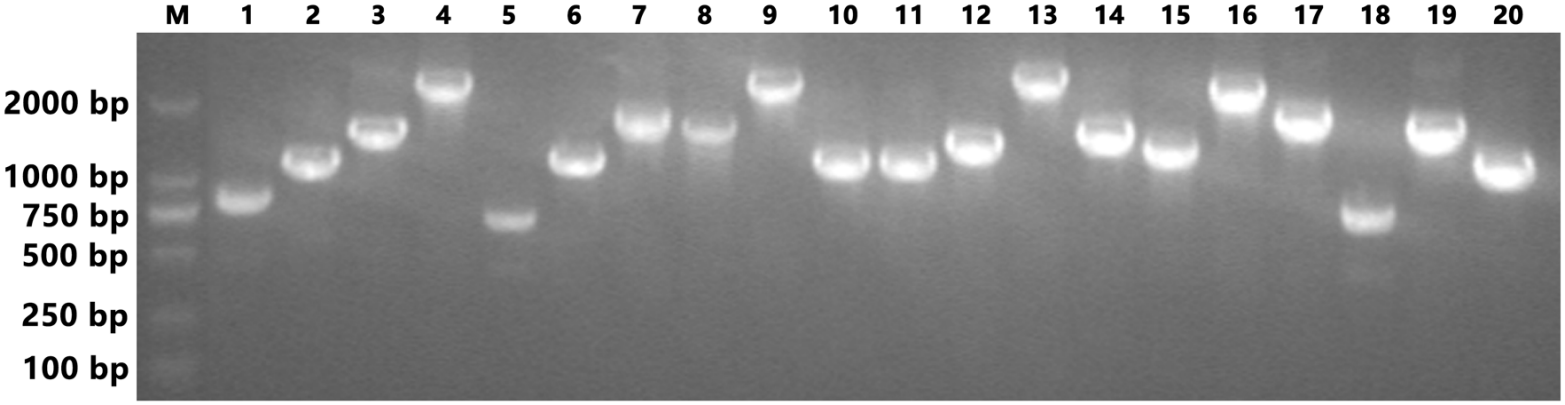
Analysis of the quality of inserts in the Y2H library. Lane M indicates the D2000 II DNA ladder (Real-Times Biotechnology), while lanes 1–20 indicate the size of insert fragments of 20 clones randomly picked from the SD/-Leu plate.

The completed Y2H library was subjected to a titer and average insert length check. Fifty microliters of a 1/1000 dilution was spread on a SD/-Leu plate, and the resulting clones (showing growth on the SD/-Leu plate) were counted after a 3 days. The plate showed 276 clones, at a titer of 1.7 × 10^7^ (276 ÷ 50 μL × 1000 × 1000 μL × 3 mL). Twenty clones were then randomly picked from the SD/-Leu plate and cultured overnight in a shaker (30°C, 250 rpm) supplied with SD/-Leu liquid medium. Plasmids were isolated from these clones, and the samples were amplified by PCR. The length of the PCR products is shown in Fig 3. The average insert length was longer than 1.2 kb. Both the titer and average length met the requirement for the Y2H screening, as mandated by the user manual of the Matchmaker^™^ Gold Yeast Two-Hybrid System.

Forty positive clones were obtained after the screening and they were sequenced. Three proteins including clottable protein, peritrophin-like protein and SPRY domain-containing SOCS box protein were identified, while the other sequences could not be annotated, possibly due to the lack of genomic information of the species.

### Confirmation of interaction between LvPT and VP37

**Fig 4:**
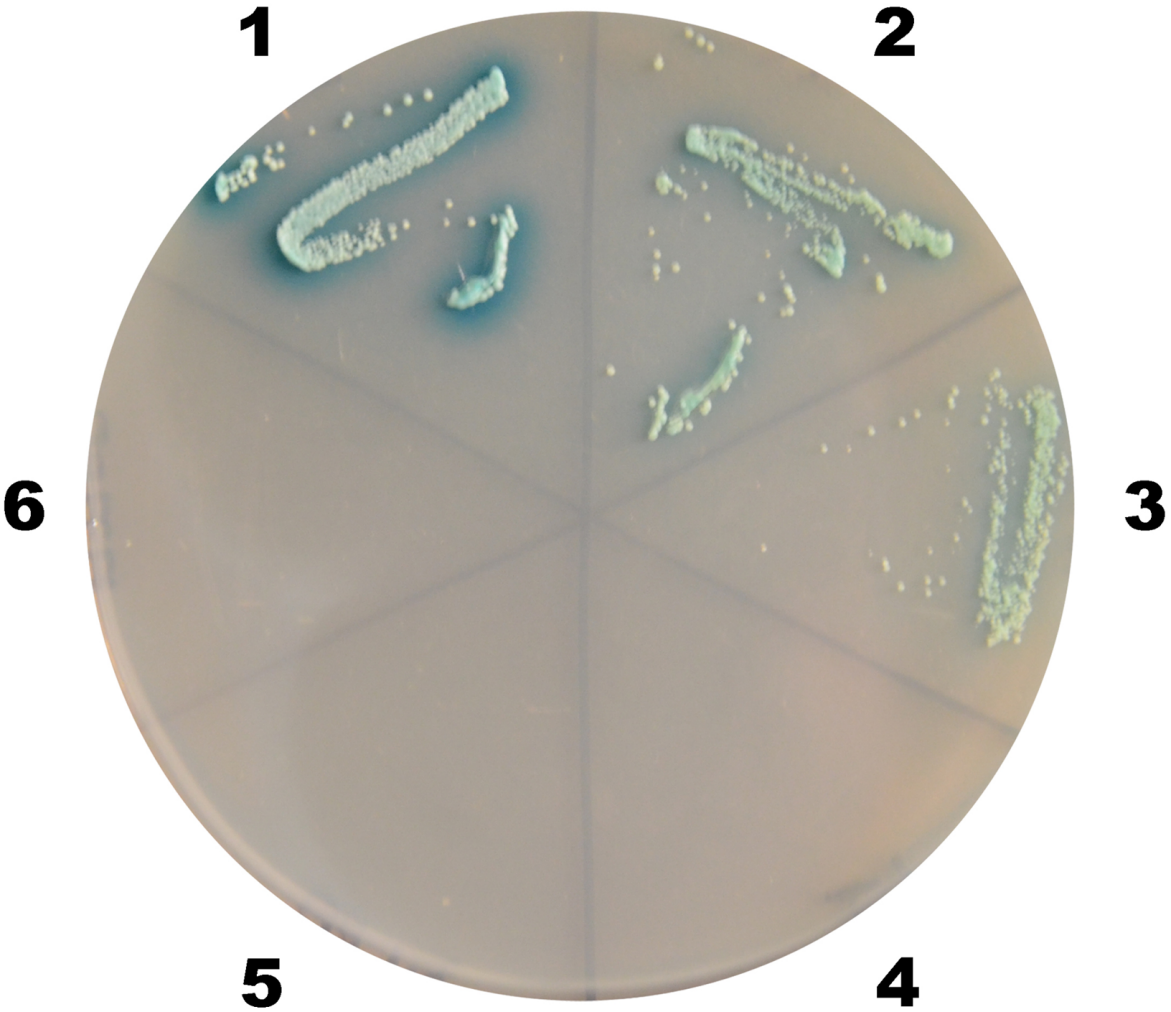
Confirmation of interaction between LvPT and VP37 on QDO/X/A plate. The blue clones on QDO/X/A plates (SD/-Ade/-His/-Leu/-Trp supplemented with X-a-Gal and Aureobasidin A) represent interactions between LvPT1/ LvPT2 and VP37. The numbers around the plate indicate the bait and prey plasmids of the transformed yeast: 1, pGBKT7-53/pGADT7-T; 2, pGBKT7-37/pGADT7-LvPT1; 3, pGBKT7-37/pGADT7-LvPT2; 4, pGBKT7-Lam/pGADT7-T; 5, pGBKT7/pGADT7-LvPT1; 6, pGBKT7/pGADT7-LvPT2

The prey plasmids and pGBKT7-37 were transformed into Y2HGold yeast in order to retest its putative interaction with VP37. Positive (pGBKT7-53/pGADT7-T) and negative (pGBKT7-Lam/pGADT7-T) controls were also prepared. The results (Fig 4) demonstrated that neither peritrophin clones (LvPT1 and LvPT2) could activate the Gal4-responsive reporters in the absence of the VP37 bait.

### Sequence analysis

**Fig 5:**
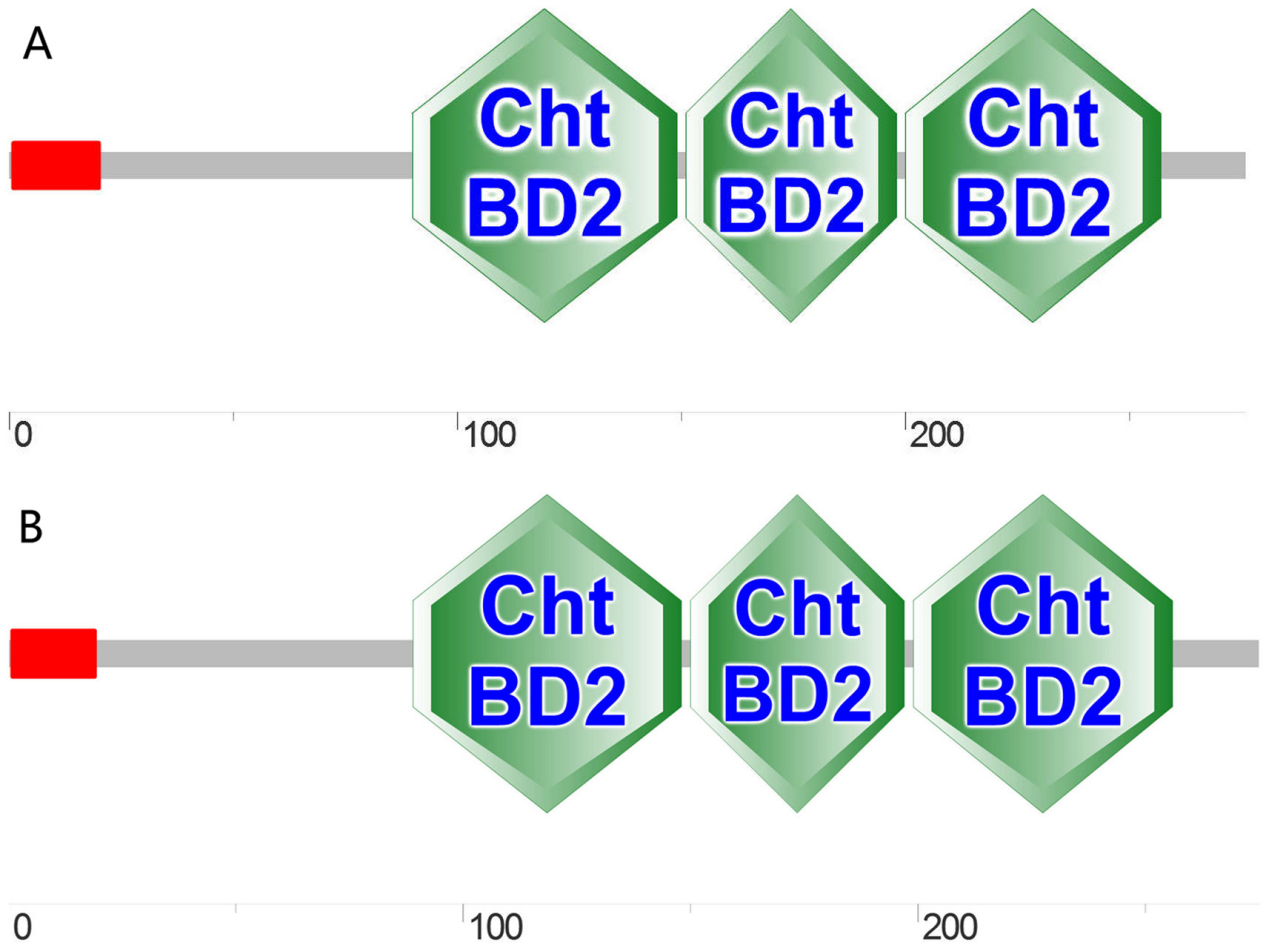
Predicted domains of LvPT1 (A) and LvPT2 (B). Amino acid sequence analysis of LvPT1 and LvPT2 revealed the presence of three conserved ChtBD2 domains; in addition, a signal peptide was observed at residues 1 to 19.

**Fig 6:**
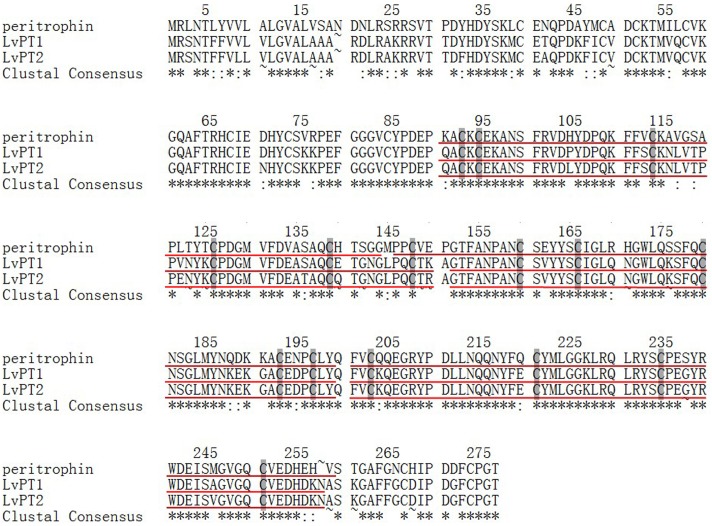
Multiple sequence alignment of LvPT1, LvPT2, and peritrophin [*Litopenaeus vannamei*]. The underlined sections indicate conserved ChtBD2 domains in LvPT1, LvPT2, and peritrophin, and the shaded sections in each ChtBD2 domain indicate the presence of cysteine residues.

The results of the protein BLAST search indicated a high identity between the amino acid sequences of LvPT1 and LvPT2 (77% and 76%) and peritrophin [*Litopenaeus vannamei*] (GenBank: AEI26265.1) (Tables 4 and 5). The nucleotide and amino acid sequence identities between LvPT1 and LvPT2 were 98% and 96%, respectively (data not shown). The predicted molecular weight (MW) and theoretical pI values of LvPT1 and LvPT2 were 30.97 and 31.05 kDa and 6.42 and 6.83, respectively. The results of protein domain prediction are displayed in Fig 5; the multiple sequence alignment performed by Clustal Omega is shown in Fig 6.

**Table 4:** BLASTP analysis of LvPT1.

Description	Total score	Query cover	E-value	Identity	Accession No.
peritrophin [*Litopenaeus vannamei*]	454	100%	3.00E-158	77%	AEI26265.1
peritrophin 3 precursor [*Penaeus monodon*]	421	97%	4.00E-145	72%	ABL86146.1
peritrophin-like protein 2 [*Penaeus semisulcatus*]	418	97%	2.00E-144	72%	AAF34332.1
peritrophin [*Fenneropenaeus chinensis*]	412	98%	8.00E-142	70%	AAZ66371.1
peritrophin-like protein 1 [*Penaeus semisulcatus*]	410	100%	6.00E-141	73%	AAF34331.1
ovarian peritrophin 1 precursor [*Penaeus monodon*]	408	99%	4.00E-140	73%	AAM44049.1
ovarian peritrophin 2 precursor [*Penaeus monodon*]	406	99%	2.00E-139	72%	AAM44050.1
cortical rod protein-2 [*Marsupenaeus japonicus*]	365	99%	1.00E-123	62%	BAD83704.1
cortical rod-like protein [*Macrobrachium rosenbergii*]	365	99%	2.00E-123	62%	BAF64507.1
cortical rod protein [*Marsupenaeus japonicus*]	363	99%	1.00E-122	62%	BAD83703.1

**Table 5:** BLASTP analysis of LvPT2.

Description	Total score	Query cover	E-value	Identity	Accession No.
peritrophin [*Litopenaeus vannamei*]	447	100%	8.00E-156	76%	AEI26265.1
peritrophin-like protein 2 [*Penaeus semisulcatus*]	416	97%	1.00E-143	72%	AAF34332.1
peritrophin 3 precursor [*Penaeus monodon*]	417	97%	3.00E-143	72%	ABL86146.1
peritrophin [*Fenneropenaeus chinensis*]	408	98%	3.00E-140	69%	AAZ66371.1
peritrophin-like protein 1 [*Penaeus semisulcatus*]	407	100%	6.00E-140	73%	AAF34331.1
ovarian peritrophin 2 precursor [*Penaeus monodon*]	403	99%	2.00E-138	71%	AAM44050.1
ovarian peritrophin 1 precursor [*Penaeus monodon*]	403	99%	3.00E-138	72%	AAM44049.1
cortical rod protein-2 [*Marsupenaeus japonicus*]	367	99%	4.00E-124	63%	BAD83704.1
cortical rod-like protein [*Macrobrachium rosenbergii*]	367	99%	5.00E-124	63%	BAF64507.1
cortical rod protein [*Marsupenaeus japonicus*]	365	99%	2.00E-123	62%	BAD83703.1

### Distribution of *LvPT* in different tissues

**Fig 7:**
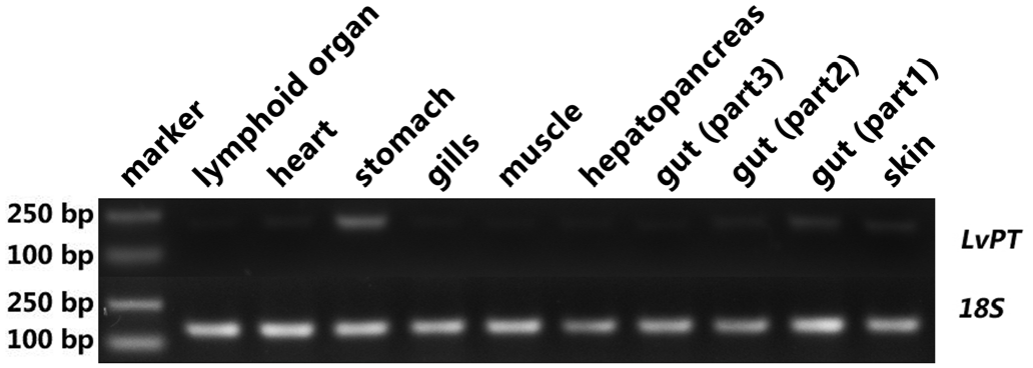
Distribution of *LvPT* in different tissues by semi-quantitative PCR. Ten tissues from 5 individuals were collected and the distribution of *LvPT* was analyzed by semi-quantitative PCR. The expected length of *LvPT* and 18S rRNA fragments were 222 and 166 bps, respectively.

Semi-quantitative PCR analysis revealed that *LvPT* was expressed in all tissues investigated. The highest expression was observed in stomach, medium expression was observed in heart, skin and gut (part1, which was most close to stomach), very weak expression was observed in other tissues (Fig 7).

### Co-immunoprecipitation

**Fig 8:**
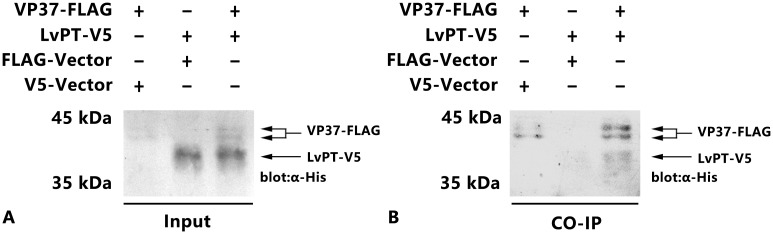
Results of co-immunoprecipitation. Sf9 cells were transfected with plasmids expressing VP37-FLAG (FLAG-tagged VP37), LvPT-V5 (V5-tagged LvPT) or empty plasmid (vector). At 6 h after heat shock, the cell lysates were harvested. (A) After separation by SDS-PAGE, expression of VP37-FLAG and LvPT-V5 was confirmed by western blot using anti-His antibody as a probe. Arrows indicate the expressed VP37-FLAG and LvPT-V5. (B) The cell lysates were immunoprecipitated with anti-FLAG M2 affinity resins and then the immunoprecipitated complexes were subjected to western blot analysis with anti-His antibody probe.

The interactions between VP37 and LvPT were investigated by co-immunoprecipitation. The results of the western blot analysis (Fig 8A) indicated that VP37-FLAG and LvPT-V5 were successfully expressed in Sf9 cells; in addition, LvPT-V5 was found to be co-immunoprecipitated with VP37-FLAG (Fig 8B).

### Expression of LvPT-V5 and LvPT^WSP^-V5 in Sf9 cells

**Fig 9:**
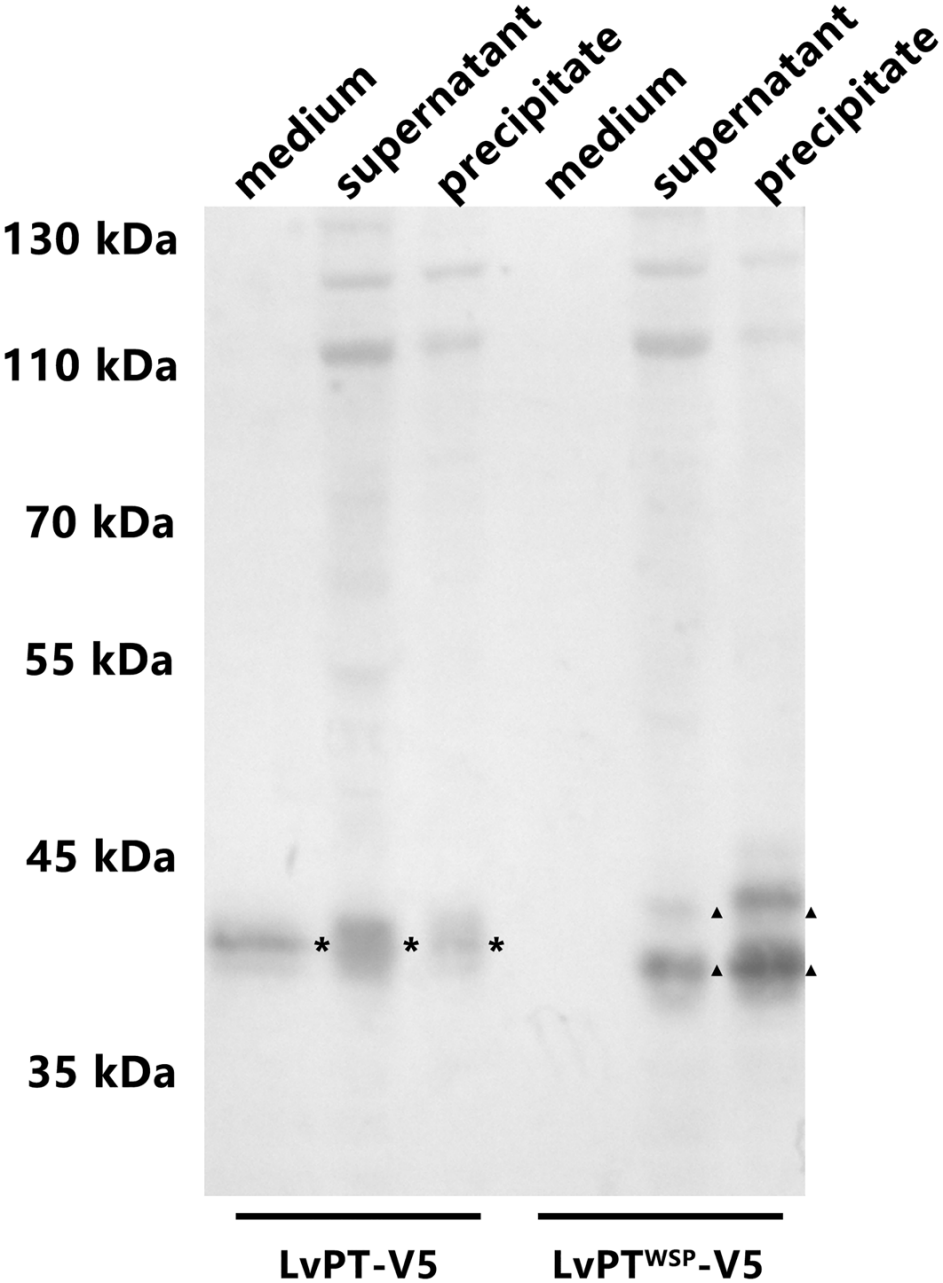
LvPT-V5 and LvPT^WSP^-V5 expression in Sf9 cells. Sf9 cells were transfected with plasmids expressing LvPT-V5 (V5-tagged LvPT) and LvPT^WSP^ -V5 (LvPT-V5 without signal peptide). At 6 h after heat shock, the culture medium, supernatant and precipitate of cell lysates were harvested. After separation by SDS-PAGE, LvPT-V5 and LvPT^WSP^ -V5 were detected by western blot using anti-His antibody as a probe. Asterisks indicate the LvPT-V5 while triangles indicate LvPT^WSP^ -V5 in samples. Compared with LvPT^WSP^-V5, LvPT-V5 could be detected in culture medium and tended to be a secretory protein in Sf9 cells.

The western blot analysis (Fig 9) revealed that both LvPT-V5 and LvPT^WSP^-V5 were expressed in Sf9 cells. In addition, LvPT-V5 expression could be detected in the culture medium, while that of LvPT^WSP^-V5 could not. This result indicated that the LvPT signal peptide could play a role in Sf9 cells, and that LvPT might be a secretory protein.

### Chitin binding assay

**Fig 10:**
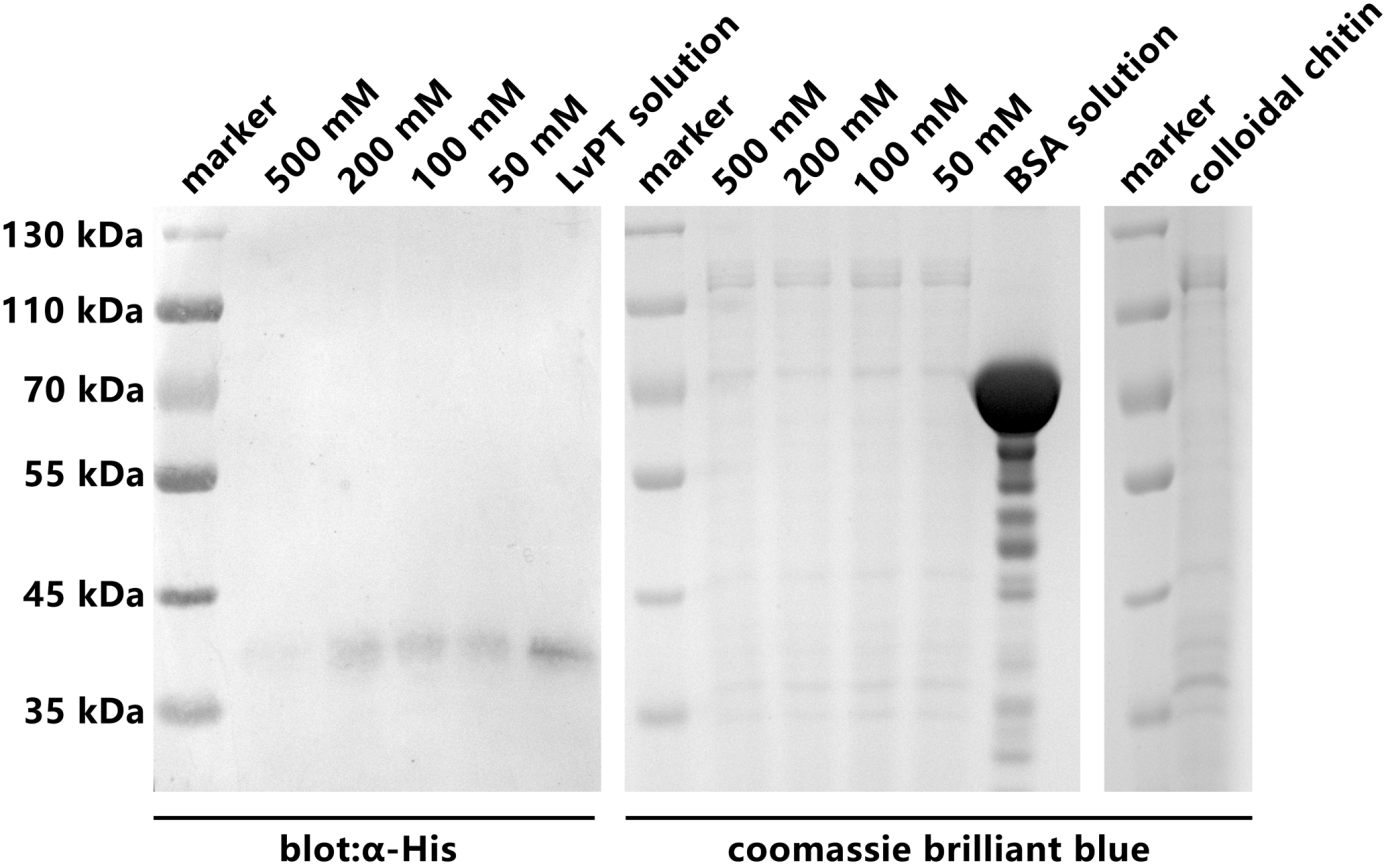
Chitin binding assay of LvPT. LvPT-V5 was incubated with colloidal chitin at 4°C for 2 h, in order to eliminate dissociative proteins, the LvPT-V5-chitin complex washed three times by TBS (50 Mm Tris, pH 8.0) supplemented with 50 mM NaCl, 100 mM NaCl, 200 mM NaCl and 500 mM NaCl, respectively. The LvPT-V5 bind to chitin (bands around 40 kDa) was detected by western blot using anti-His antibody as a probe. BSA was used as negative control and detected by coomassie brilliant blue. Colloidal chitin (protein-free) was used as reference to indicate the bands in negative control were not proteins but polymer of N-acetyl glucosamine.

The results of the chitin binding assay (Fig 10) showed that LvPT-V5 could specifically bind to colloidal chitin. The binding of LvPT-V5 to chitin persisted after washing thrice with TBS containing 200 mM, 100 mM, and 50 mM NaCl. In fact, LvPT-V5 could be detected even after the LvPT-V5-chitin complex was washed with TBS containing 500 mM NaCl. In contrast, BSA could not be detected after washing the BSA-chitin complex with TBS containing a low concentration of NaCl.

### Interaction between LvPT and WSSV envelope proteins

**Fig 11:**
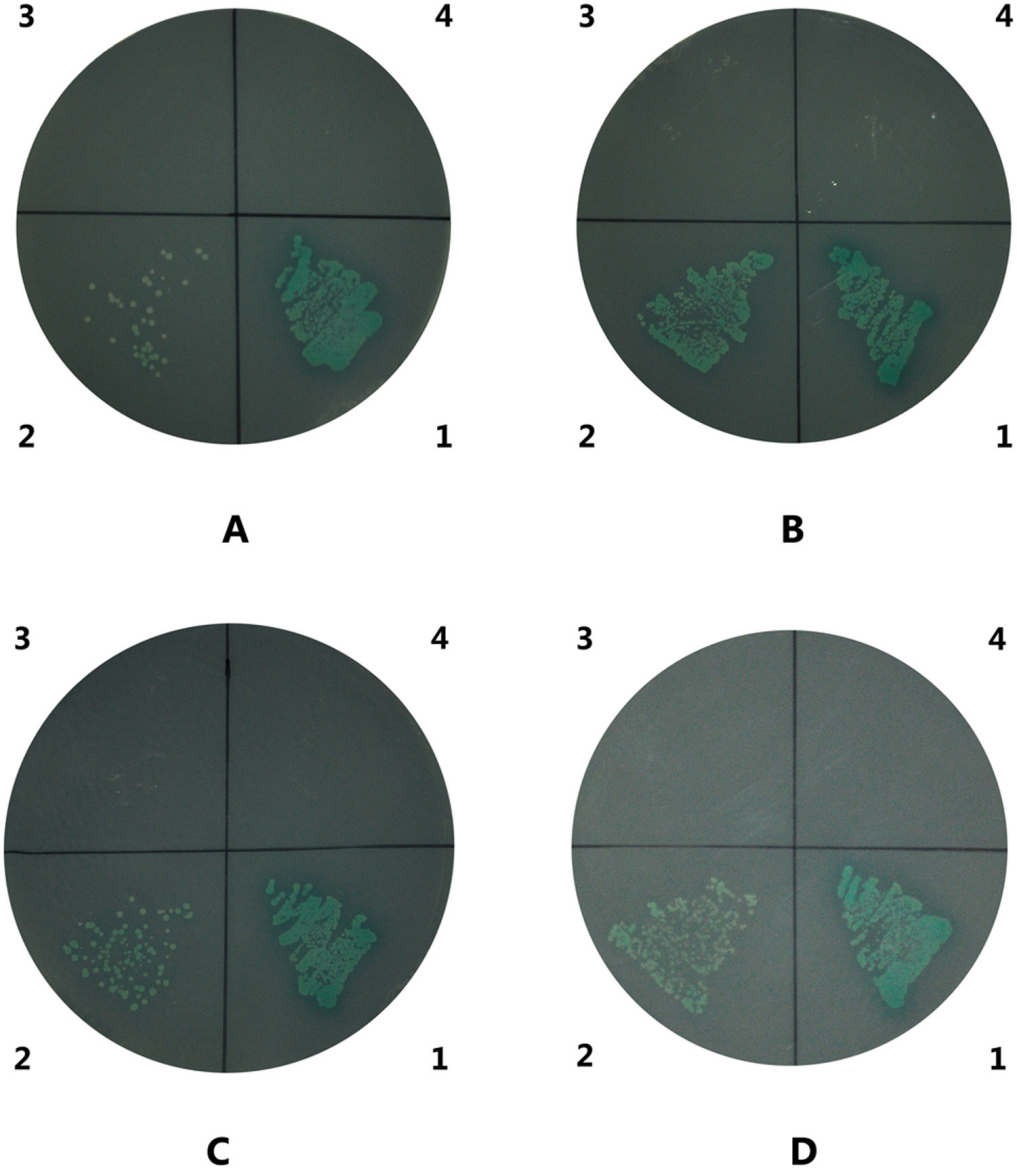
Interactions between LvPT and VP32, VP38A, VP39B, and VP41A. The blue clones on QDO/X/A plates (SD/-Ade/-His/-Leu/-Trp supplemented with X-a-Gal and Aureobasidin A) represent interactions between LvPT and VP32 (A), VP38A (B), VP39B (C), and VP41A (D). The numbers around the plate indicate the bait and prey plasmids of the transformed yeast: 1, pGBKT7-53/pGADT7-T (positive control); 2, pGBKT7-VPXX/pGADT7—LvPT1; 3, pGBKT7/pGADT7-LvPT1; 4, pGBKT7-Lam/pGADT7-T (negative control).

The results of this analysis revealed that LvPT1 could also interact with VP32, VP38A, VP39B, and VP41A (Fig 11). The interaction of VP38A with LvPT was stronger than that of VP32, VP39B, and VP41A. However, the other envelope proteins mentioned in Table 2 did not show any significant interaction with LvPT.

## Discussion

In this study, a Y2H library was successfully constructed using cDNA synthesized from the genetic material in *L*. *vannamei* stomach and gut tissues. The titer and average insert length of this library were found to be suitable for screening. VP37 is a WSSV envelope protein that is located on the outside of the virion in the currently accepted three-dimensional model of the WSSV membrane protein complex [15]. A previous study indicated that VP37 played a major role in WSSV infection [16]. Recombinant VP37 has shown a high-binding activity with the shrimp cell membrane in a binding assay [17]. Therefore, VP37 was believed to be involved in WSSV infection in the host stomach and gut. As the full length of VP37 could autonomously activate all reporter genes in Y2H system, a truncated C-terminal VP37 was used as bait in the Y2H screening.

Following screening, sequence analysis, and positive confirmation of the obtained clones, we identified two peritrophin clones that could interact with VP37 and activated the Gal4-responsive reporter genes in the Y2H system. However, these two clones showed high nucleotide and amino acid sequence similarity. As the tissue samples used for Y2H library construction were collected from two individuals, the differences between LvPT1 and LvPT2 were attributed to differences between the individuals. Therefore, *LvPT1* and *LvPT2* were considered to be the same gene *LvPT*. SMART analysis revealed the presence of a signal peptide at the N-terminal and three ChtBD2 domains in LvPT, denoting a high similarity with other shrimp peritrophin proteins. Co-immunoprecipitation confirmed the interaction between VP37 and LvPT. An additional Y2H experiment also identified interactions between LvPT and VP32, VP38A, VP39B, and VP41A. Although a previous study [18] reported that VP38A and VP41A were auto-activated, we observed no such auto-activation in VP38A or VP41A in our analyses, which was similar to the results of another study [19] (where VP41A did not show any auto-activation). This difference could be attributed to the differences in yeast strains, culture conditions, and analysis methods.

Peritrophin is a structural protein expressed on the peritrophic membrane (PM); it is listed among the third class of PM proteins [20]. PM is a non-cellular structure that lines the gut of many arthropods and other animals, thereby separating the ingested food from the gut epithelium [21]. In *L*. *vannamei*, PM compartmentalizes the gut lumen, separating the ingested material from the epithelial tissue; therefore, PM is a potential early site of entry for pathogens (bacterium, fungi, or virus) [22]. The results of the tissue distribution analysis revealed that LvPT was mainly expressed in the stomach (compared to other tissues, including the gut). Following transfection, both LvPT-V5 and LvPT^WSP^-V5 were expressed by Sf9 cells. However, only LvPT-V5 could be detected in the supernatant of the culture medium; this confirmed the function of the predicted signal peptide at the N-terminus of LvPT. The chitin binding assay revealed that LvPT-V5 could bind to chitin. Previous studies have indicated that chitin is a major component of the PM. These results suggested that LvPT may be a secretory protein mainly expressed in the stomach, which plays a role in the formation of PM. A previous study of the PM in *Sicyonia ingentis*, which has characteristics similar to those of the PM of *L*. *vannamei*, reported that only particles smaller than 20 nm could physically pass through the membrane [23]. However, the size and length of WSSV is 70–150 nm (at its broadest point) and 250–380 nm, respectively [24], which is much larger than the permissible limit for permeating the PM in *S*. *ingentis*. WSSV can infect the host via the oral route; in fact, the epithelial cells in the midgut trunk have been considered as a transient site of WSSV infection [9,25]. Therefore, there must be receptors or assisting proteins in the stomach and gut that can enable the WSSV to break through the physical barrier of the PM.

VP37 was found to interact with LvPT via Y2H screening and co-immunoprecipitation. Further analyses between LvPT and other envelope proteins of WSSV revealed that VP38A, VP39B, and VP41A could also interact with LvPT. Previous studies have indicated that shrimp proteins containing a chitin-binding domain are involved in the immune response. The *Penaeus monodon* chitin-binding protein (PmCBP) in *P*. *monodon* interacts with the envelope protein of WSSV [19]. Peritrophin-like proteins (EcPT) in *Exopalaemon carinicauda* are also believed to be involved in WSSV infection. In fact, Wang et al. observed a decrease in mortality compared to the control group under WSSV challenge following the silencing of *EcPT* expression by dsDNA interference [26]. Therefore, we believe that our findings in combination with the findings of previous studies could help elucidate the mechanism by which WSSV infects the host through the oral route.

Although this study identified an interaction between the peritrophin-like protein from shrimps and WSSV envelope proteins for the first time, the progress of WSSV infection through the oral route and its key factors remain to be elucidated. Our future research will focus on elucidating the mechanism by which WSSV takes advantage of LvPT during infection.
